# Postoperative Capsular Bag Distension Syndrome: Case Report of an Early Case Following Athalamia due to Vigorous Eye Rubbing

**DOI:** 10.1155/crop/8798298

**Published:** 2026-05-30

**Authors:** Lilian Demolin, Elie Motulsky, Ayoub El Belhadji, Oscar Kallay

**Affiliations:** ^1^ Ophthalmology Department, Hôpital Universitaire de Bruxelles, Brussels, Belgium; ^2^ Faculty of Medicine, Université Libre de Bruxelles, Brussels, Belgium, ulb.ac.be; ^3^ Ophthalmology Department, Centre Médical de l’Alliance, Braine-l’Alleud, Belgium

## Abstract

**Introduction:**

Capsular bag distension syndrome (CBDS) is a rare complication of phacoemulsification, characterized by fluid retention in the capsular bag leading to reduced visual acuity, a myopic shift, and a risk of pupillary block.

**Case Presentation:**

We report the case of a 65‐year‐old male who developed CBDS after rubbing his eye vigorously postoperatively. On Postoperative Day 1, his best corrected visual acuity (BCVA) was 0.1 with a myopic shift. We made the diagnosis of CBDS with the Scheimpflug camera and AS‐OCT. Conservative management was initially attempted, but due to persistent symptoms, surgical aspiration of the trapped fluid was performed on Day 4. This intervention resulted in BCVA improvement to 0.8 and resolution of the myopic shift.

**Discussion:**

Differential diagnoses considered included incorrect IOL power, IOL misplacement, transient corneal edema, and incisional astigmatism. We hypothesize that mechanical athalamia due to eye rubbing was an acute trigger leading to forward IOL displacement, adhering it to the anterior capsule and trapping fluid. A stepwise management approach, including observation followed by surgical intervention, proved effective.

**Conclusion:**

CBDS may be precipitated by transient mechanical athalamia following vigorous eye rubbing, which can promote acute intraocular lens–capsular apposition and fluid entrapment. This case highlights the importance of postoperative patient education. Early recognition and management are essential to prevent severe complications such as endophthalmitis and pupillary block.

## 1. Introduction

Capsular bag distension syndrome (CBDS) is a rare but clinically significant complication following phacoemulsification with intraocular lens (IOL) implantation in the capsular bag. It is characterized by the accumulation of fluid between the posterior capsule and the IOL optic, resulting in anterior displacement of the lens and a consequent myopic shift.

CBDS has been described in both the early and late postoperative periods. Early‐onset CBDS, typically occurring within the first days after surgery, has most commonly been attributed to retained ophthalmic viscosurgical device (OVD), particularly sodium hyaluronate, trapped behind the IOL optic due to a valve‐like apposition between the anterior capsule and the lens surface [1–4]. In contrast, late‐onset CBDS is associated with the accumulation of turbid or milky fluid within the capsular bag, sometimes referred to as “lacteocrumenasia,” and has been linked in some cases to low‐grade infection with *Propionibacterium acnes* [4–8]. Reported risk factors include residual viscoelastic material, incomplete cortical cleanup, postoperative inflammation, and a small capsulorhexis.

Management strategies differ depending on the timing and clinical presentation. In early CBDS, initial conservative management with close observation may be appropriate, particularly in cases with mild symptoms. However, persistent myopic shift or patient dissatisfaction may necessitate intervention, including surgical aspiration of the trapped fluid or Nd:YAG laser capsulotomy, either posterior or, as suggested by some authors, anterior [8, 9].

In contrast, late‐onset cases are more commonly managed with Nd:YAG laser posterior capsulotomy [10], although a surgical approach should be considered when capsulotomy is not feasible or when infection is suspected. In such cases, pars plana vitrectomy combined with posterior capsulotomy and aspiration of the capsular contents allows both therapeutic management and microbiological or histopathological analysis [11, 12].

## 2. Case Presentation

A 65‐year‐old male underwent clear lens extraction for refractive purposes, with bilateral implantation of multifocal IOLs. His medical history included mild right eye (RE) amblyopia due to anisometropia. Preoperative best corrected visual acuity (BCVA) was 0.4 in the RE and 1.0 in the left eye (LE), with a manifest refraction of +4.25 (−0.50) 144° RE and +2.25 (−0.25) 26° LE.

The RE was operated on first, and a +25.50 D AT LISA tri839 MP (Zeiss) hydrophilic multifocal IOL was implanted without intraoperative complications. A high‐viscosity cohesive OVD (NuVisc PRO, BVI) was used during the procedure.

On Postoperative Day 1, the patient reported blurred distance vision with preserved near vision. He reported having rubbed his RE vigorously in the shower despite postoperative instructions. BCVA in the RE was 0.1 (20/200), with a refraction of −3.25 (−0.25) 102°, and intraocular pressure (IOP) was 6 mmHg. Slit‐lamp examination revealed anterior chamber narrowing and mild inflammation (Tyndall 1+) without the Seidel sign. Fundus examination showed a normal retina, with no evidence of choroidal detachment or retinal folds. The patient was advised to avoid any ocular manipulation, to wear a protective eye shield continuously, and to continue topical steroidal, nonsteroidal anti‐inflammatory, and antibiotic therapy.

**Figure 1:**
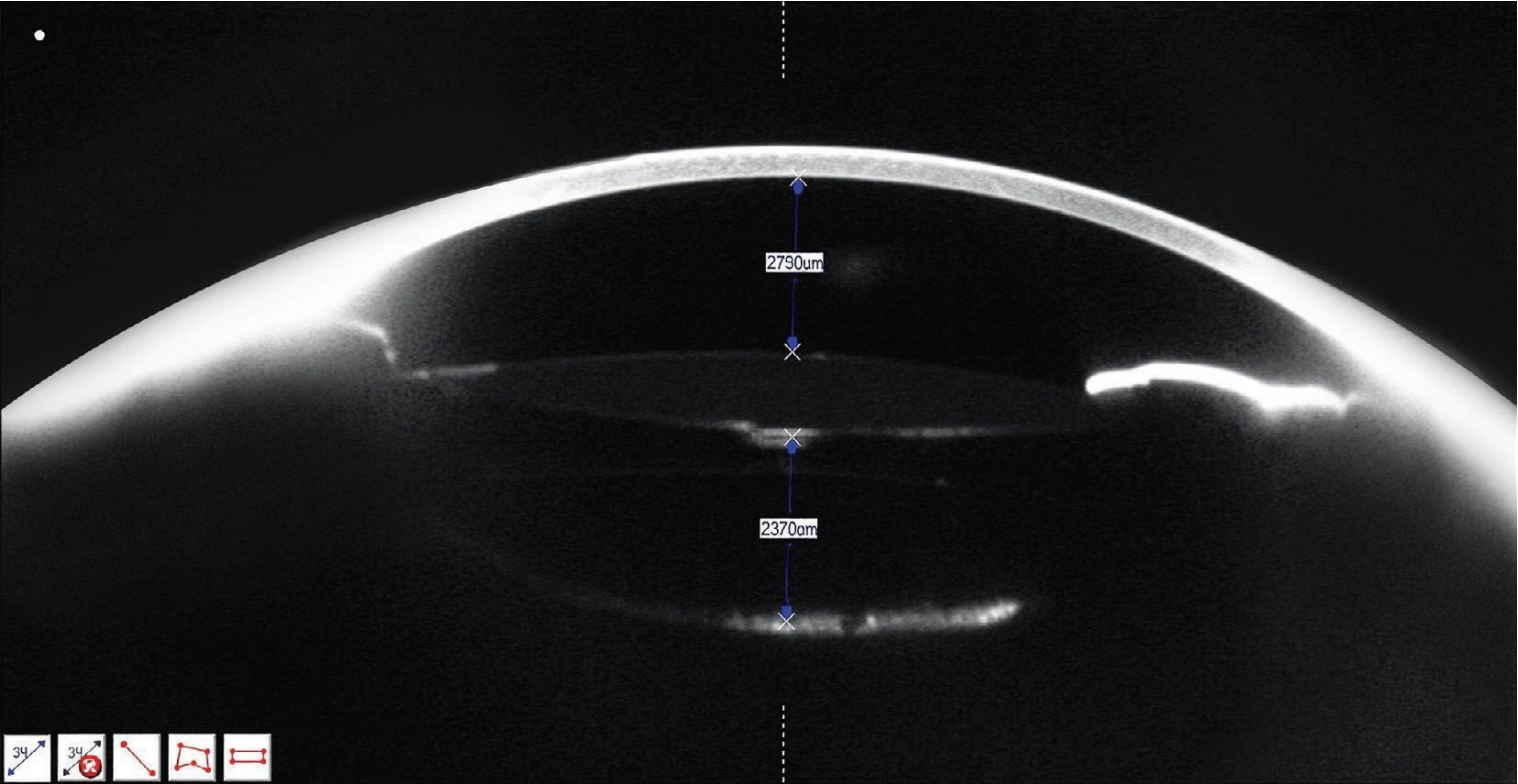
Scheimpflug camera (Pentacam HR; Oculus Inc.) imaging of RE showing the distension of the capsular bag on Postoperative Day 2. ACD: 2790 *μ*m; distance between the posterior surface of the IOL and the posterior capsule: 2370 *μ*m.

**Figure 2:**
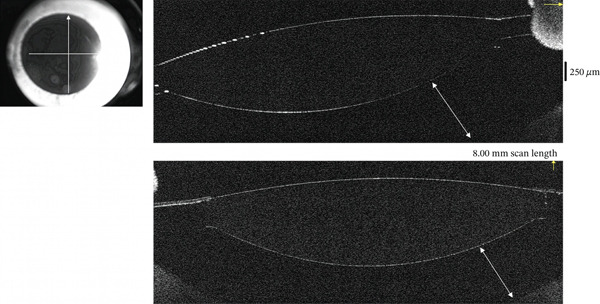
AS‐OCT imaging of OD showing distension of the capsular bag on Postoperative Day 2. Arrows show the distension of the bag.

At Postoperative Day 2, BCVA improved to 0.2 (20/100), with a refraction of −2.50 (−1.00) 113°. The anterior chamber depth had returned to normal. However, the IOL appeared anteriorly displaced, with posterior capsular bag distension. Pharmacologic pupil dilation confirmed that the IOL haptics remained within the capsular bag. Scheimpflug imaging (Pentacam HR; Oculus Inc.) and anterior segment OCT (RTVue XR Avanti, Optovue Inc.) demonstrated anterior displacement of the IOL and capsular bag distension with optically clear material behind the lens (Figures 1 and 2 and Table 1).

**Table 1:** Scheimpflug camera (Pentacam HR; Oculus Inc.) measurement in preoperative assessment and Postoperative Days 2 and 5.

	Preoperative assessment	Postoperative Day 2	Postoperative Day 5
Anterior chamber depth (ACD)	2340 *μ*m	2790 *μ*m	3330 *μ*m
IOL to posterior capsule distance	N/A	2370 *μ*m	Nonmeasurable

These findings were consistent with early postoperative CBDS. Conservative management was initiated, including close observation, cycloplegic drops, and continuation of topical therapy.

On Postoperative Day 3, BCVA was 0.3, with stable refraction and an IOP of 10 mmHg. Fundus examination remained normal.

Given the persistence of the myopic shift and the patient′s visual symptoms, surgical intervention was performed on Postoperative Day 4. The capsular bag was irrigated and aspirated to remove the trapped fluid.

**Figure 3:**
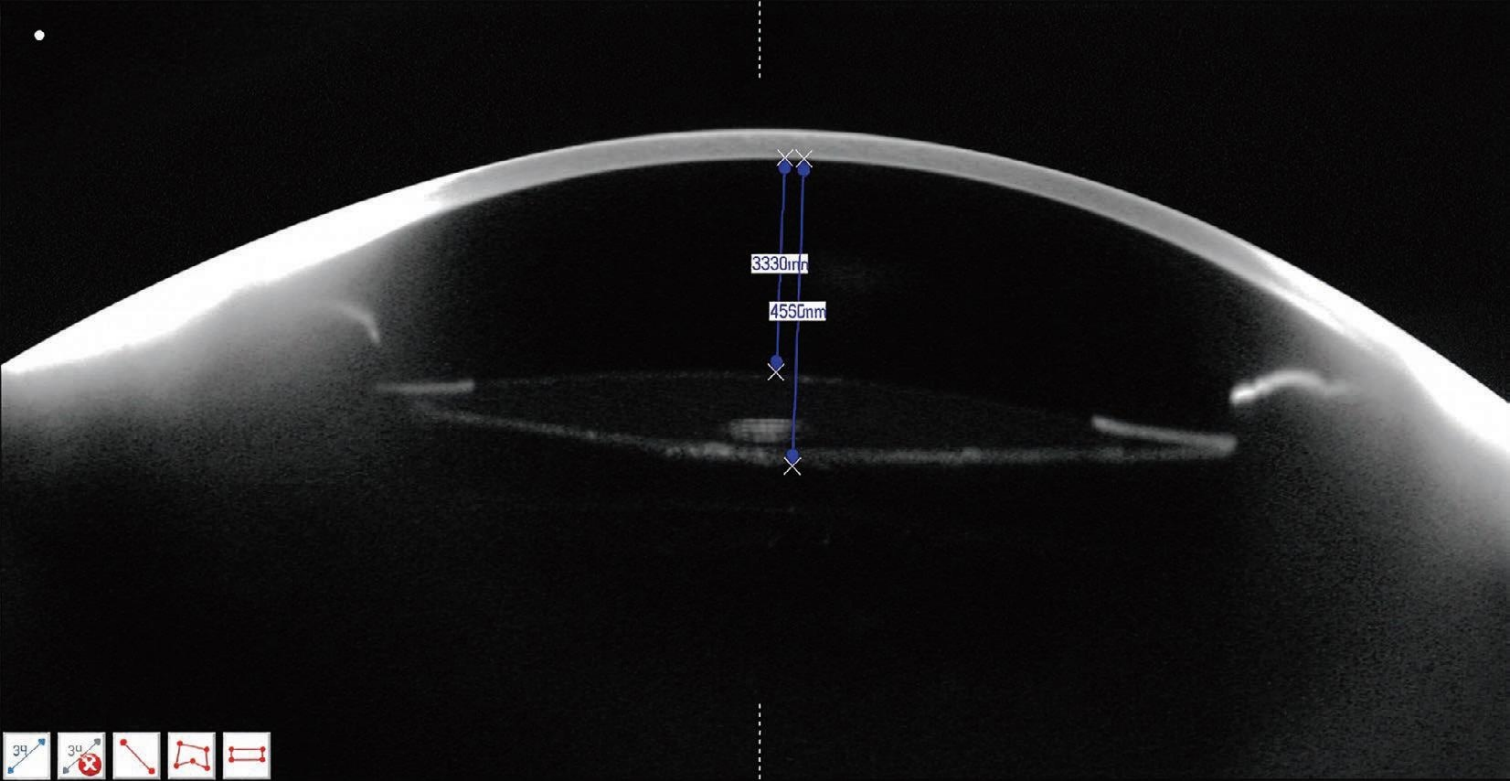
Scheimpflug camera (Pentacam HR; Oculus Inc.) imaging of the RE showing the normal capsular bag on Postoperative Day 5. ACD: 3330 *μ*m; distance between the posterior surface of the IOL and the posterior capsule: nonmeasurable.

**Figure 4:**
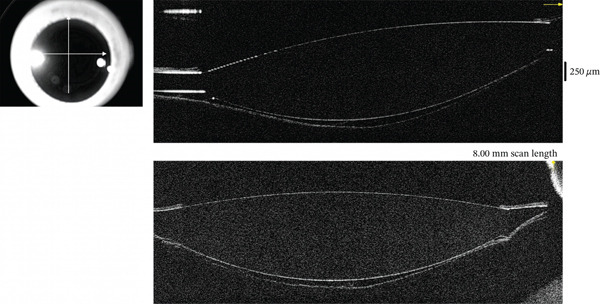
AS‐OCT imaging of the RE showing a normal capsular bag on Postoperative Day 5.

On Postoperative Day 5, BCVA improved to 0.8 (20/25), with a refraction of −0.75 (0) 0°. Repeat Scheimpflug and anterior segment OCT imaging showed normalization of the capsular bag configuration (Figures 3 and 4 and Table 1).

Surgery on the LE was subsequently performed without complications, and no recurrence of CBDS was observed in either eye.

## 3. Discussion

Diagnosis was established via slit‐lamp examination, Scheimpflug imaging, and AS‐OCT. Differential diagnoses included incorrect IOL power calculation, IOL misalignment, transient corneal edema, or incisional astigmatism. However, the anterior IOL displacement and capsular distension suggested CBDS.

We started by checking the IOL calculation using True Barrett, the IOL choice using the medical record in order to confirm that the correct IOL was inserted, and the IOL alignment was evaluated at the slit‐lamp exam. Then, the IOL subluxation in the sulcus was ruled out by slit‐lamp inspection after pupil dilation. The multifocal IOL (+25.5 D) and its haptics were well positioned and centered within the capsular bag. The continuous curvilinear capsulorhexis measured approximately 5.5 mm, which is within the optimal range. These findings allow us to reasonably exclude known anatomical risk factors for CBDS, such as an oversized capsulorhexis or IOL decentration. Although the viscoelastic device was carefully removed at the end of surgery, the presence of small residual amounts cannot be completely excluded.

We acknowledge that early CBDS is classically attributed to retained viscoelastic material trapped behind the IOL, facilitated by a “valve‐like” apposition between the anterior capsule and the IOL optic. However, in typical postoperative cases, this configuration develops gradually in a relatively stable anterior chamber environment [3].

In contrast, we propose that, in this case, vigorous eye rubbing induced a transient mechanical athalamia, resulting in acute anterior chamber collapse and a sudden drop in anterior segment pressure. This acute event may have led to a forward displacement of the IOL, promoting an immediate and firm apposition between the IOL optic and the posterior surface of the anterior capsule. We hypothesize that this abrupt and forceful contact differs from the gradual sealing described in conventional early CBDS, potentially creating a more effective barrier to fluid egress. Under these conditions, even minimal residual viscoelastic material could become trapped within the capsular bag. The combination of low IOP and tight capsule–IOL adhesion may have prevented its clearance. Subsequently, an osmotic gradient could have driven aqueous humor into the capsular bag, leading to its distension. In addition, postoperative inflammation may have further reinforced the seal between the anterior capsule and the IOL surface.

Therefore, rather than excluding retained viscoelasticity as a contributing factor, we propose that mechanical athalamia acts as a precipitating event that facilitates and amplifies the well‐described mechanism of early CBDS. The key distinction in this case lies in the acute, externally triggered collapse of the anterior chamber, which may create conditions that are not typically present in routine postoperative scenarios.

To our knowledge, the potential role of transient mechanical athalamia as a trigger for CBDS has not been previously described. While this hypothesis remains speculative, it highlights a possible additional pathway in the pathophysiology of CBDS and warrants further investigation.

Although Nd:YAG laser posterior capsulotomy is a recognized treatment option for CBDS, it was deliberately avoided in this case. In the context of clear lens extraction with multifocal IOL implantation, this approach is irreversible and may be associated with undesirable complications such as floaters and dysphotopsia, particularly relevant in patients with high refractive expectations [13]. A stepwise management strategy was therefore adopted, initially consisting of medical therapy and close observation. However, despite normalization of anterior chamber depth, the patient demonstrated persistent myopic shift, stable capsular bag distension on imaging, and ongoing visual dissatisfaction. These findings suggested that spontaneous resolution was unlikely in the short term, prompting surgical intervention on Postoperative Day 4. Irrigation and aspiration of the capsular bag allowed controlled removal of the entrapped material while preserving capsular integrity and restoring the normal anatomical configuration. Notably, the aspirated fluid appeared more aqueous and lacked the typical cohesive characteristics of retained viscoelastic material. This observation may indicate that fluid accumulation was not solely attributable to residual viscoelastic but could also involve secondary aqueous inflow into the capsular bag, supporting a multifactorial mechanism in this case.

An uneventful cataract surgery for clear lens exchange is not always a smooth journey. In this present case, two important complications have been avoided. First, due to the anterior positioning of the IOL, we feared the risk of a pupillary block. To prevent this, we decided to keep the patient dilated under cycloplegic drops. Another serious potential complication was the development of endophthalmitis, as the retained fluid could serve as a culture medium for microorganisms. The association between *Propionibacterium acnes* infection and postoperative CBDS has been well documented [11, 12]. Additionally, the patient′s RE had undergone surgery twice within 4 days. Therefore, close postoperative follow‐up was crucial to ensure that the intraocular inflammation remained under control. A thorough postoperative care education, with an accent on the importance of not rubbing eyes, remains a major step for the success of the surgery.

## 4. Conclusion

CBDS is a rare but potentially serious postoperative complication following uneventful cataract surgery or clear lens exchange. While retained viscoelastic material remains a well‐established contributing factor, this case suggests that transient mechanical athalamia—induced by vigorous eye rubbing—may act as a precipitating event by promoting acute IOL–capsular apposition and facilitating fluid entrapment within the capsular bag.

This underscores the importance of postoperative patient education, particularly regarding the avoidance of eye rubbing, as well as adherence to meticulous surgical technique, including appropriate capsulorhexis sizing and thorough viscoelastic removal. Early recognition and a stepwise management approach, including both medical and surgical interventions, were essential for successful resolution. Given the potential complications, such as pupillary block and endophthalmitis, prompt diagnosis and timely treatment remain critical to achieving favorable visual outcomes.

## Funding

No funding was received for this manuscript.

## Consent

Patient consent was obtained.

## Conflicts of Interest

The authors declare no conflicts of interest.

## Data Availability

Data sharing is not applicable to this article as no datasets were generated or analyzed during the current study.
